# Mobile Health Augmented Cardiac Rehabilitation (MCard) in Post-Acute Coronary Syndrome Patients: A randomised controlled trial protocol

**DOI:** 10.12669/pjms.37.3.3664

**Published:** 2021

**Authors:** Aliya Hisam, Zia Ul Haq, Zohaib Khan, Patrick Doherty, Jill Pell

**Affiliations:** 1 Dr. Aliya Hisam, MBBS, MPH, FCPS, PhD Scholar. Department of Community Medicine Army Medical College, NUMS, Rawalpindi, Pakistan; 2 Prof Zia Ul Haq, MBBS, MPH, PhD, Department of Public Health & Social Sciences, Khyber Medical University, Peshawar, Pakistan; 3 Dr. Zohaib Khan, MBBS, PhD, Department of Public Health & Social Sciences, Khyber Medical University, Peshawar, Pakistan; 4 Dr. Patrick Doherty, PhD. Department of Health Sciences, University of York, United Kingdom; 5 Prof. Jill Pell, MBChB, MD, FFPH. Institutes of Health & Wellbeing, University of Glasgow, United Kingdom

**Keywords:** Acute coronary syndrome, Cardiac rehabilitation, Communication, Health-related quality of life, Mhealth, Mobile health, Randomised controlled trial, Rehabilitation, Text messages

## Abstract

**Objectives:**

To determine the effectiveness of mobile health augmented cardiac rehabilitation (MCard) on health-related quality of life (HRQoL), clinical and behavioural outcomes in post-ACS.

**Methods:**

A single-centre, single-blinded, two-arm randomised controlled trial is planned at Armed Forces Institute of Cardiology (AFIC), Pakistan. The duration was two years, that is from January 2019 till December 2020. A total of 160 participants were recruited and randomly allocated to the control group or the intervention group. Intervention is a mobile health augmented cardiac rehabilitation (MCard), a medically supervised cardiac rehabilitation program for 23-24 weeks. The phase one includes individual counselling during the hospital stay and in phase two includes communication of standardised messages related to healthy lifestyle modification through a specifically designed software.

**Results:**

This clinical trial results will give insight into the impact of MCard in improving the health outcomes (HRQoL, clinical and behavioural) of participants. If proven to be effective, this technology can be scaled up and implemented in other cardiac centres in the country. It utilises fewer human resources and can be delivered at a lower cost.

**Conclusion:**

The study protocol will be giving evidence either MCard can contribute to improving the HRQoL, clinical and behavioural outcomes of post-ACS patients following hospital discharge. Considering the COVID-9 situation, this is the perfect time to implement and evaluate the effectiveness of MCard on health outcomes among post-ACS patients.

## INTRODUCTION

Non-communicable diseases (NCDs) and their risk factors have emerged as one of the major health problems globally.^1^ A significant contributor to NCD mortality is cardiovascular diseases; most notably acute coronary syndrome (ACS).^2^ Cardiovascular disease incidence and mortality are falling in high-income countries. Still, in contrast, they are rising in low and middle-income countries where they now account for nearly half of the disease burden.^3^

ACS has a significant impact on patients’ physical and psychological well-being, contributing to the deterioration of their health-related quality of life (HRQoL). Impaired HRQoL leads to poor health outcomes in the form of morbidity or mortality.^4^ Cardiac rehabilitation has been adopted in the cardiac institute to deal with post-ACS patients’ physical and mental well-being. Many clinical studies^5^,^6^ has proven the effectiveness of cardiac rehabilitation. Still, access, acceptability and affordability to these cardiac rehabilitation programs are limited with overall participation between 20 per cent to 50 per cent only.

Comprehensive cardiac rehabilitation programs as secondary preventive measures involving personalised counselling sessions, behaviour change modification, and empowering patients with self-monitoring devices can reduce the ACS associated with premature mortality, morbidity, and healthcare costs.^7^

Novel ways^8^ are now being explored to improve the provision and uptake of cardiac rehabilitation. Mobile technology provides affordable access to many patients at low cost and without travel and distance constraints. Therefore, there is a growing interest in its potential role in health promotion for modifiable risk factors prevalent in ACS patients.

A clinical trial has been devised to implement a telecommunication based cardiac rehabilitation in which participants will be encouraged for self-care. Suppose this intervention proved to be effective in terms of bringing a change in their HRQoL, behavioural and clinical outcomes, then in coming years. In that case, mhealth technology could be a convenient and effective way of communicating health messages to post-ACS patients in health care settings.

## METHODS

This clinical trial’s study objective is to assess a cardiac rehabilitation program’s effectiveness in which text messages to post-acute coronary syndrome (post-ACS) patients related to healthy lifestyle modification will be communicated. The objectives was as follows:

1. To determine the effectiveness of MCard on HRQoL outcomes in post-ACS patients.
2. To determine the effectiveness of MCard on clinical outcomes in post-ACS patients.
3. To determine the effectiveness of MCard on behavioural outcomes in post-ACS patients.
4. To determine the acceptability of the MCard among post-ACS patients.

We hypothesised that post-ACS patients who receive the interventions would positively impact their health outcomes (HRQoL, clinical and behavioural) compared to those who receive the usual care. This paper is written per the Standard Protocol Items: Recommendations for Interventional Trials (SPIRIT) 2013 statement. The trial will be registered in line with CONSORT 2010 Statement: updated guidelines for parallel reporting group randomised trials. The detail of participants enrollment with a trial schedule is described through the Table-I and the SPIRIT checklist is attached as supplementary file-1.

**Table-I:** Schedule of enrollment, intervention and assessment of recruited participants according to SPIRIT 2013

		Study Period (In weeks)			

	Enrolment	Baseline	Allocation	Post-allocation	
TIMEPOINT		1-2		12	24
***ENROLMENT:***					
Eligibility screen	X				
Informed consent	X				
Empowering with Self-monitoring devices & brochure to fill		X			
Allocation			X		
***INTERVENTIONS:***					
*[Group A]*				⟷	
*[Group B]*				⟷	
***ASSESSMENTS:***					
*Basic Demographic Variables*		X			
HRQoL (MacNew QMLI & SF-12)		X		X	X
*Clinical Outcomes*		X		X	X
*Behavioural Outcomes*		X		X	X
*Adherence*					X

This will be a single-centre, single-blinded, two-arm randomised controlled trial conducted at Armed Forces Institute of Cardiology (AFIC). The clinical trial is registered with the Australian New Zealand Clinical Trials Registry (ANZCTR) under trial identity number ACTRN12619001731189.^9^

The total study duration will be approximately two years from January 2019 till December 2020. After analysing hospital data, it is expected that patient recruitment will take approximately 16-20 weeks (at least 10-12 patients per week) while intervention will take 23-24 weeks.

The patient coming to AFIC emergency and admitted in its wards with ACS diagnosis with ACS will be enrolled in this clinical trial after fulfilling the eligibility criteria as in Table-II. ACS will be diagnosed by at least one on-call cardiologist (cardiac consultant) based on the chest or epigastric pain, radiating to the arm, jaw, neck or back), cardiac markers ((Creatine phosphokinase and/or creatine kinase-MB and/or Troponin I)) and/or electrocardiogram (ST elevation or non-ST elevation).

**Table II:** Inclusion and exclusion criteria for MCard

Inclusion criteria	Exclusion criteria
i.Age eligibility: From 35 years till 65 years.	i.Mini-mental State Examination of less than 24.
ii.Gender eligibility: Both	ii.Not physically or mentally capable of handling a mobile phone.
iii.Diagnosed with documented ACS evidence by a cardiologist based on the chest or epigastric pain, radiating to the arm, jaw, neck or back and/or cardiac markers and/or electrocardiogram.	iii.Diagnosed with familial hypercholesterolemia.
iv.Ejection fraction of >25%.	iv.History of cerebrovascular accident.
v.Patients are having mobile phone ownership or a caretaker who can assist in handling mobile.	v.Patient on hemodialysis or peritoneal dialysis.
vi.Patients are giving fully informed voluntary written consent for participation.	vi.A patient diagnosed with hepatitis B or C by serology.
vii.The patient may only have received medical treatment or may have undergone cardiac revascularisation procedure percutaneous coronary intervention or thrombolytic therapy.	vii.Concurrent severe disease, such as malignancy.
	viii.Is or may be pregnant.

The primary outcome measure that is HRQoL physical component summary was used for sample size estimation in STATA version 14. A sample size of 61 per group in a ratio of 1:1 was calculated by two independent mean tests of SF-36 physical component (control group; 39.09 and intervention group 42.65),^10^ assuming SF-36 physical component variability of 6.74 and 7.14 in control and intervention group respectively, a power of 80% and a significance level of 5%. To allow for a predicted 25% drop-out or lost to follow-up rate from adverse events, the sample size has been increased to 80 per group, recruiting 160 participants (80 x 2=160) altogether.

After recruitment into the clinical trial, the baseline information will be recorded by a data collector. The data collector will be blinded to the participant’s actual trial arm before and after the intervention. The random assignment of recruited participants into the two arms was performed by a research associate using web-based permuted block randomisation (4 x 8), ensuring 1:1 ratio between the two arms (i.e. 80 in each). Allocation concealment will be conducted by the sequentially numbered opaque sealed envelope.

The data collector will be communicating with the participants (intervention and control group) for the follow-up data collection at 12 weeks and 24 weeks. Participants will be requested not to disclose their counselling session or text message communication to the data collector. At 12 and 24 weeks, the participants will be asked for a follow-up to the hospital. If the patient cannot come for follow-up, the information will be collected via telephone interview within the ± 01 week period of 12 or 24 weeks.

### Intervention Group:

MCard will be a medically supervised cardiac rehabilitation program for a total period of 23-24 weeks for the intervention group. The Phase-I will be initiated during the hospital stay and will include one individual face to face behavioural change modification counselling using HEARTS, a technical package for cardiovascular disease management by the World Health Organization.^11^ Phase-II will be initiated after hospital discharge. The intervention details are devised in line with Tidier guidelines^12^ and are attached as supplementary file 2. The details of the groups are described in Table-III.

**Table III:** Detail of the cardiac rehabilitation intervention of the two groups

	Phase I (During hospital stay)			Phase II (After discharge)

	Standard post-ACS care	Empowering with self-monitoring devices	Behaviour change modification counselling	Mobile Text communication
Group A (n=80, control)	√	√		
Group B(n=80, intervention)	√	√	√	√

The core component of the MCard is that the participants will receive the same standardised messages on their mobile phones related to healthy lifestyle modification through a specifically designed software. This will be two-way communication in which the patient will receive text messages and inquire info through texting back. A health professional (principal investigator) and / or an associate researcher will be receiving the calls and messages from the patients and respond to them. The mobile text messages will be the same for all the intervention group. Manually one message per day will be communicated to group B for 23-24 weeks. The principal investigator will communicate a total of 161-168 messages through the MCard designed software. The health messages have been devised by first having a small group discussion with patients to identify the gap in knowledge related to modifiable risk factors (physical inactivity, unhealthy diet, salt intake, tobacco use and medicine defiance). The devised messages were rephrased for communication after having a consultation with a cardiologist. Rephrasing of the messages was conducted for easy understanding of the participants.

### Control Group:

The control group will receive standard care. However, they will not receive text messages on their mobile related to healthy lifestyle modifications.

### Self-monitoring devices and brochure:

The standard post-ACS care and self-monitoring devices (digital blood pressure machine, digital weight machine and a pedometer) was provided to all patients of both arms to determine the actual impact of health messages communication. All the participants will be briefed on how to use the above three devices. They will be made practically aware of how and when to use these devices**.** Participants will be asked to fill in the brochure their blood pressure and early morning empty stomach body weight once in a week while steps taken shall be recorded daily at the end of the day before they go to sleep.

A trained research associate will assess participants three times during the whole study period that is 23-24 weeks first at baseline before they are discharged from hospital after taking informed written consent. Then at 12 weeks, at the follow-up period and then at 24 weeks post-discharge. In case follow-up is missed or is not undertaken, the telephonic interview will be undertaken. The participant’s demographic questionnaire will be inquired, including information regarding age, gender, educational status, medical history, income per month, the preferred medium of text communication, and behavioural and clinical parameters. At the end of the 24^th^ week, along with other information, the MCard program’s subjective acceptability will also be evaluated on a Likert scale.

### Primary Outcome is HRQoL:^13^

It is a multidimensional concept that describes individuals or groups’ perceived physical and mental health over some time. The primary outcome changes in HRQoL which will be assessed by validated disease-specific (MacNew quality of life after myocardial infarction (MacNew QLMI) form)^14^ and generic instruments (Short Form 12 (SF-12)).^15^

### Secondary Outcomes:

- ***Clinical Outcomes:*** Clinical outcomes are quantifiable changes in physical health as a result of health care interventions. In this randomised control trial, the following clinical outcomes will be recorded:
  - ***Physiological parameters:***
    * Blood pressure measurement (systolic and diastolic in millimetres of mercury) will be taken according to the Centers for Disease Control and Prevention.
    * Body mass index measurement will be derived from weight in kilogram divided by height in meters squared.
  - The number of days of current hospitalisation at 12 and 24 weeks if any.
  - Number of readmissions to the hospital within 180 days after ACS date.
  - Major adverse cardiovascular events (MACE): After hospital discharge, if patients come to the hospital with repeat-MI, heart failure, symptom-driven coronary revascularisation or death, they will be identified as having a MACE.
- ***Behavioural outcomes:*** It refers to the end result of action to improve their health. The following patient behavioural outcomes will be assessed on the subjective basis within the last four weeks:
  - Self-monitoring (systolic and diastolic blood pressure in millimetres of mercury and body weight in kilogram at least once weekly)
  - Number of steps taken per day measured by pedometer (Physical activity)
  - ***Medicine compliance:*** Taking medicines according to their cardiologist prescription.
  - ***Salt restriction:*** Subjective patient salt intake will be recorded. They will be asked if they think they are taking less than one teaspoon per day in all food.
  - ***Smoking status (no smoker, current smoker or ex-smoker):*** Smoking status of the patient’s last month will be recorded.
  - ***Healthy diet preferences:*** Subjective patient healthy diet will be recorded. They will be asked what type of food they prefer. Are they following the recommended diet?
  - ***Acceptability:*** At the end of the clinical trial, at 24 weeks, the post-ACS patient will be asked to judge the program acceptability on a Likert scale from 1 to 5, 1 being unacceptable and 5 being acceptable.

Data will be entered and analysed in STATA version 14 using the intention to treat principle. Descriptive data will be presented as frequency and percentages. Continuous data will be presented by means with their 95% confidence intervals. Table and graphs will be constructed for reporting sociodemographic, behavioural and clinical data. Age, gender, education level, comorbidity will be included as potential covariates. All of the analyses will be taken statistically significant at a two-tailed p-value of < 0.05.

### Ethics approval and consent to participate:

The Ethical Review Committee of Khyber Medical University (DIR/KMU-EB/MH/000486) and Armed Forces Institute of Cardiology (AFIC-IERB-SOP-15) has approved the clinical trial protocol and its amendments before the start of the study. Written informed consent was taken from each participant before enrolling them.

## RESULT

Ethical and institutional permission has been sought. Research associate has been hired, and their training is also completed. Participants recruitment has been planned, and the primary data completion date is December 2020. After that, the results will be analysed and expected to be published within three months of work completion.

## DISCUSSION

Pakistan is a resource-constrained country whose health care system faces the triple burden of diseases (i.e. communicable diseases, non-communicable disease and injuries). Our health care system is a vulnerable one and is trying to deal with this emerging burden of NCDs precisely the increasing burden of cardiovascular disease.^16^ Tobacco, physical inactivity, unhealthy diet are the modifiable risk factors which if are not controlled in post-ACS patients, increases their risk of re-infraction along with increased mortality and morbidity.^17^,^18^

A comprehensive preventive intervention that is adopted by many developed countries for coronary artery diseases is cardiac rehabilitation. The primary purpose of the cardiac rehabilitation programs is to maximise patients’ physical functionality, improve their quality of life, and prevent the recurrence of major cardiovascular and cerebrovascular events.^19^

Clinical guidelines strongly recommend that patients have access to a cardiac rehabilitation program, and there is strong scientific evidence of its effectiveness. However, globally, the overall participation in these cardiac rehabilitation programs ranges only between 20 per cent to 50 per cent.^14^ In these cardiac rehabilitation studies,^20^ HRQoL is often used as an outcome measure and is a known predictor of post-ACS patient’s morbidity and mortality.

Studies^21^-^23^ conducted in developed and developing countries targeting mobile health communication having different intervention period starting from one month to two years with averaging six months duration. In developed countries, websites, mobile application, and other smart communication have been created to incorporate mhealth into health care setup.

The MCard is one of the first clinical trials conducted in Pakistan, which has incorporated two-way communication using mobile technology modifiable risk factors to impact post-ACS patients’ outcomes, i.e. HRQoL, clinical and behaviour. One of the study limitations is that it is a single centred study, if further studies are to be planed, they shall be multi-centred as it would be portraying a wider picture od the trial.

## CONCLUSION

There has not been a single randomised controlled trial conducted in Pakistan, which involved mobile health communication in cardiac rehabilitation. Considering the COVID-9 situation, this is the perfect time to implement and evaluate the effectiveness of MCard on health outcomes among post-ACS patients.

### Authors’ contributions:

**AH** conceived and designed trial design, data acquisition and analysis, drafting work. Responsible and accountable for the accuracy or integrity of the work

**ZUH**: Conception, design of the work and manuscript revision critically.

**ZK:** Design of the work and editing of final manuscript.

**PD, JP**: Review and final approval of the manuscript to be published.
